# Posteriorly displaced salter halter fracture-dislocation at the sternoclavicular joint with associated thoracic outlet syndrome: A case report

**DOI:** 10.1016/j.ijscr.2020.06.025

**Published:** 2020-06-11

**Authors:** Timothy McAleese, Mark Curtin, Denis Collins

**Affiliations:** a National University of Ireland, Galway, Co. Galway, Ireland; b Department of Trauma and Orthopaedics, Beaumont Hospital, Dublin, Ireland

**Keywords:** Posterior sternoclavicular joint dislocation, Physeal fracture-dislocation, Salter-Harris 2, Thoracic outlet syndrome, Conservative management, Case report

## Abstract

**Introduction:**

Posterior sternoclavicular joint fracture-dislocations are a rare and often missed diagnosis. They represent <1% of shoulder girdle injuries and are nine times less common than anterior dislocations. These injuries can be associated with life-threatening complications such as compression of the superior mediastinal structures including the great vessels and brachial plexus.

**Presentation of Case:**

This case describes a 23-year-old woman who was initially discharged from the emergency department but represented 8 days later with symptoms of venous and neurogenic thoracic outlet syndrome as a result of posterior displacement of a Salter 2 fracture-dislocation at the sternoclavicular joint. Multidisciplinary consensus and patient preference resulted in the conservative management of her injuries with intensive rehabilitation and close outpatient follow-up.

**Discussion:**

The evidence regarding this rare injury is evolving. It currently suggests all posteriorly displaced fracture-dislocations at the sternoclavicular joint are reduced. Closed reduction is often unsuccessful and open reduction is high risk and must be undertaken in the presence of a cardiothoracic surgeon which may not always be appropriate or in line with patient preferences. There are limited reports of successful conservative management of these injuries and none in the setting of thoracic outlet syndrome.

**Conclusion:**

This unique case report is the first to describe outcomes of a conservatively managed, posteriorly displaced fracture-dislocation at the sternoclavicular joint with associated venous and neurogenic thoracic outlet syndrome. This information will benefit select patients.

## Highlights

• Posterior sternoclavicular joint fracture-dislocations are a rare and often-missed injury in trauma.
• Posterior displacement at the SCJ is a true emergency and can be associated with compression of vital structures and thoracic outlet syndrome.
• Closed or open reduction of these injuries is generally advised but is associated with considerable risk.
• Conservative management can be successful in the presence of physeal injury but has never been described in the setting of thoracic outlet syndrome.

## 1 Introduction

Posterior sternoclavicular joint (SCJ) fracture-dislocations are an extremely rare traumatic injury representing <1% of shoulder girdle injuries. They are nine times less common than anterior dislocations [1]. The physis of the clavicle represents a weak point vulnerable to fracture or traumatic epiphyiolysis in patients under the age of 25 as it is the last to fuse in the body [2].

Posterior displacement at the SCJ is a true emergency and can be fatal if missed due to the vital structures that lie posterior to the medial clavicle. Superior mediastinal structures such as the brachial plexus, great vessels, trachea and oesophagus are only separated by the strap muscles and can suffer simple compression or more serious injuries [3]. This can manifest as thoracic outlet syndrome (TOS) which is characterised by a variety of neurovascular symptoms resulting from maladjustment between a specific anatomical space and the neurovascular bundle conveyed to the arm.

The appropriate management of SCJ fracture-dislocations is under constant review owing to the infrequency of these injuries although it is currently recommended that all true dislocations are reduced to negate the impending risk of mediastinal compromise and to avoid future shoulder pain and loss of function. However, closed reduction is often unsuccessful and open reduction internal fixation (ORIF) is associated with significant surgical risk and may not be appropriate or desired by all patients [4]. There are limited reports of conservative management in young patients with physeal injury and none in the setting of TOS [5,6].

Upholding the SCARE guidelines, this unique case describes the outcomes of a conservatively managed, posteriorly displaced fracture-dislocation at the sternoclavicular joint associated with venous and neurogenic thoracic outlet syndrome [7].

## 2 Presentation of case

**Fig. 1:**
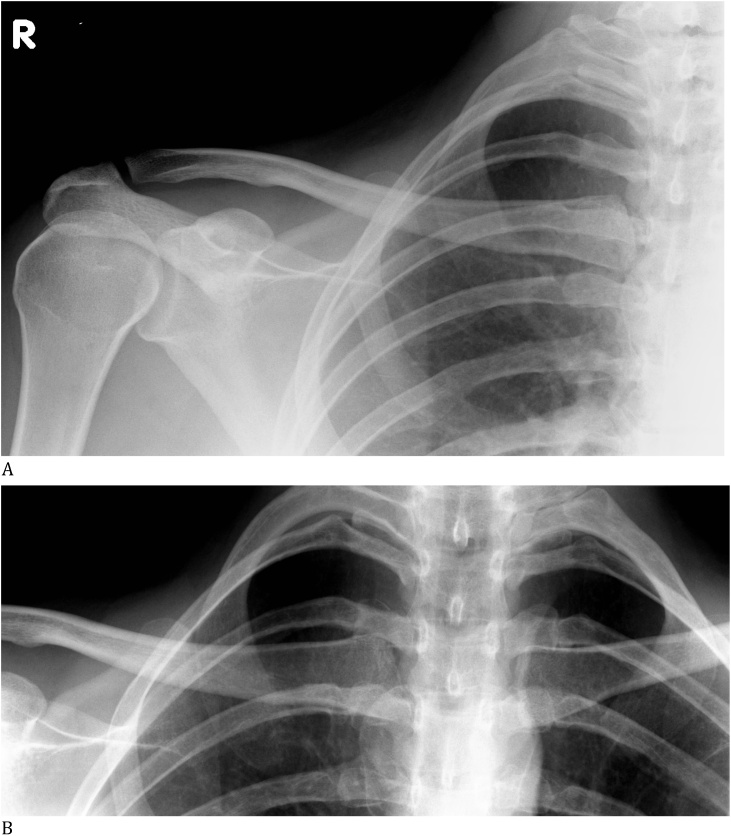
**A:** AP radiograph of right clavicle on presentation which showed no obvious fracture or dislocation of the shoulder girdle. **B:** AP radiograph of bilateral sternoclavicular joints showed subtle joint space widening on the right side. The overlapping of medial structures such as the ribs, clavicle, sternum and vertebrae impedes visualisation of the sternoclavicular joint in direct antero-posterior films.

A 23 year old woman self-presented to the emergency department with a swollen, painful proximal clavicle and decreased range of motion in her right shoulder. She had sustained indirect trauma to her SCJ from a blow to the lateral aspect of her clavicle during internal rotation of the arm. On exam, she was neurovascularly intact, her initial X-rays were negative and she was discharged from the emergency department in a sling, managed as an occult, undisplaced proximal clavicle fracture (Fig. 1A & B).

One week later, she represented with uncontrolled medial clavicular pain associated with intermittent paresthesia of her ipsilateral forearm and fingers and weakness in the intrinsic muscles of her right hand. These symptoms worsened with repetitive movements and with raising her arm above her head. She also reported episodes of spontaneous right upper limb swelling associated with a grey discolouration. She found that holding her shoulder forward and tilting her head to the side of the dislocation produced partial alleviation of the pain. She also mentioned that supporting the ipsilateral arm reduced the neurological symptoms and allowed the power in her hand return. She reported no dysphagia, dyspnea, hoarseness or claudication symptoms. Her past medical, surgical and family history were unremarkable and she took no regular medications.

On exam her blood pressure was 143/86, heart rate 70 beats/min, temperature was 36.6 °C, respiratory rate 16 breaths/min and pulse oximetry 98 % on room air. Her medial clavicle was swollen without evidence of subcutaneous emphysema and her range of motion was reduced in all planes due to pain inhibition. Her neurological exam demonstrated altered sensation to her forearm and hand and weak grip strength. Finger abduction, adduction and extension were preserved, as were all movements of the thumb. Her cap refill time was appropriate and her radial and brachial pulses were palpable.

**Fig. 2:**
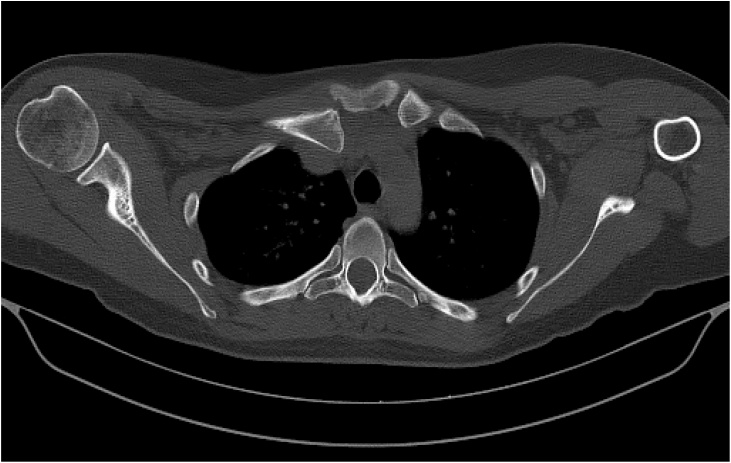
Axial CT without contrast at the level of the joint revealed a Salter Harris 2 fracture with posterior displacement at the right sternoclavicular joint. There was a small fragment of bone at the superior-anterior aspect of the joint represents the ossifying epiphysis. The relationship of the medial clavicle to the subclavian vein and brachiocephalic vein may is seen by subsequent CT with intravenous contrast.

**Fig. 3:**
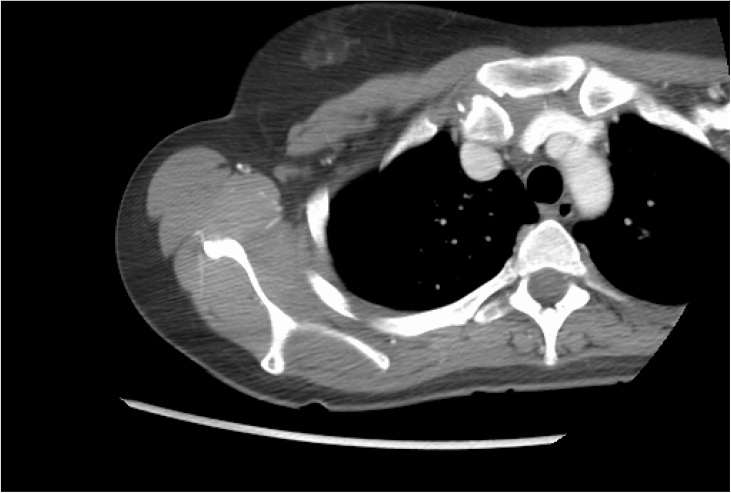
CT angiogram with the patients right arm above their head showed the medial end of the clavicle was posteriorly displaced as noted previously. There was no evidence of any vascular compression, either arterial or venous. The subclavian, axillary and brachial arteries were also normal.

Axial CT Thorax showed posterior dislocation of the SCJ with potential compression of the subclavian and brachiocephalic vein. A fracture line was noted anteriorly in keeping with a salter harris 2 epiphyseal fracture (Fig. 2). CT angiography was performed with the arm abducted above the head to out rule dynamic compression and showed mild compression of the axillary-subclavian junction between the clavicle and first rib although this was deemed positional (Fig. 3).

Our patient was subsequently discussed at a multidisciplinary meeting. Considerations were made to the degree of joint displacement on the CT, the lack of radiographic compression, the operative risk of open reduction after a delayed presentation, the increased remodeling potential in the presence of physeal fracture and our patient’s preference. Conservative management was advised, although close outpatient follow-up for worsening symptoms was recommended. Our patient, who was keen to avoid surgery, agreed to clinical follow-up with oblique radiographs every 3 months for 1 year while attending outpatient physiotherapy. She subsequently undertook SC joint protective and shoulder stabilising rehabilitation until her symptoms had improved, followed by progressive range of motion and strengthening exercises.

**Fig. 4:**
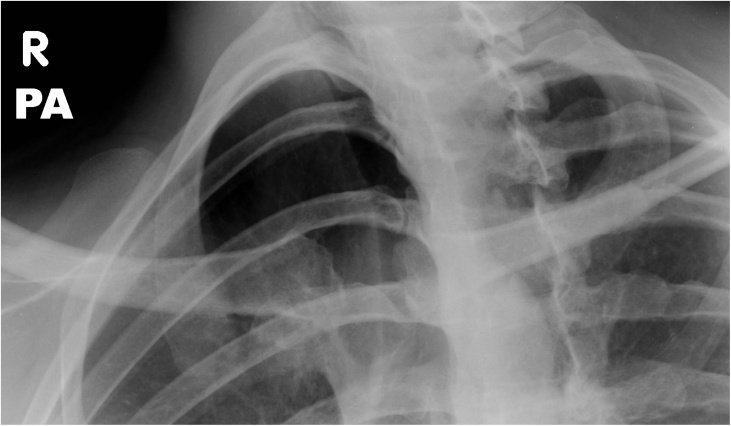
XR at 12 months follow-up. Best seen on the right oblique radiograph, the anatomic relationship of the medial aspect of the right clavicle and the right sternum is abnormal with abnormal widening but unchanged compared to the previous XR. This reflects stable posterior SCJ fracture-dislocation.

Her most recent X-ray showed her SCJ remains dislocated but stable (Fig. 4). She is currently 3 years post-injury and her symptoms have completely resolved other than mild venous congestion after extensive, repetitive overhead movements. Full range of motion has returned in her shoulder and she reports her injury has little impact on her quality of life. Her Oxford Shoulder Score is 44/50.

## 3 Discussion

Posterior dislocations at the SCJ represent <1% of shoulder girdle injuries and are nine times less common than anterior dislocations [1]. They are misdiagnosed in 50 % of patients, owing to the rarity of the condition and the subtlety or delay in clinical presentation [8].

The sternoclavicular joint is a multiaxial, synovial saddle joint that is stabilised by an anterior and posterior joint capsule and 4 ligaments: anterior and posterior sternoclavicular, costoclavicular, interclavicular. Posteriorly, the ligaments are a lot stronger making anterior dislocation a lot more common. The physis at the medial clavicle is the last in the body to fuse and remains a weak point until it fully ossifies at the age of 22−25. This coupled with increased joint laxity makes sternoclavicular joint dislocation and displaced physeal fracture more common in younger patients.

Plain radiographs are often not adequate for diagnosis. Oblique views such as the Hobbs axial view, the Heinig or the Kattan view can be slightly more sensitive but require specialist radiological interpretation and knowledge of an uncommon injury [9]. CT is the gold standard for detecting these injuries. It is more sensitive and ensures prompt diagnosis of concomitant physeal fractures. Intravenous contrast is recommended to detail mediastinal injuries or vascular compression. Pre and post-reduction venograms may be useful when venous structures are involved to measure venous pressure and flow at various points and to assess reduction adequacy [10].

When deliberating over the appropriate management of SCJ displacement, the severity of potential acute and chronic complications should be considered. Acutely, compression and laceration of the subclavian and brachiocephalic vessels have been described along with secondary haemo-pneumothorax and damage to the trachea and oesophagus. These injuries can be fatal [4]. Brachial plexus neuropathy such as in our case is also possible. Importantly, long-term complications also occur. Mehta et al. describe a patient who developed subclavian artery compression after 6 months, mandating medial clavicle excision [11]. Stankle et al. highlight two patients, the first remained asymptomatic for 2.5 years before developing mild cramping and cyanosis and the other developed subclavian vein thrombosis after 4.5 years. Other long-term complications include tracheo-oesophageal fistula formation, early-onset post-traumatic arthritis and brachial plexus traction injuries from chronic shoulder abduction [12].

The appropriate management of SCJ fracture-dislocations and the timing of intervention is a cause of ongoing dispute owing to the infrequency of these injuries. The current evidence-base includes a limited number of case series and case reports but recommends all posterior fracture-dislocations are reduced to negate the risk of mediastinal compromise and to avoid future shoulder pain and loss of function [4]. However, closed reduction only has a 68 % success rate and this remarkably decreases if not undertaken within 48−72 hrs of injury [13,14] or if attempted in the presence of physeal fracture as opposed to true dislocation. Lafosse et al. described the failure of closed reduction in all cases of posteriorly displaced physeal fractures and half of true dislocations [15]. Eskola et al. report 8 cases of closed reduction, of which 5 re-dislocated due to inherent joint instability [16]. Closed reduction is also not without complications [17]. Waters et al. describe 13 cases where 2 of their initial 3 cases had to be taken to theatre for ORIF after failed closed reduction indicating how failed closed reduction may necessitate open reduction which may be high risk. There are increasing recommendations for ORIF when closed reduction fails, for recurrent instability or any patients with neurovascular symptoms [15,18]. However, operative management is associated with significant surgical risk and it is advised a cardiothoracic surgeon is present. There are several techniques successfully described in case reports but no fixation has been shown superior to another, although is widely agreed that hardware such as Balser plates are to be avoided due to the risk of injury to the medial physeal plate, as should fixation with Kirschner wires which can migrate [19,20].

Conservative management has been successfully described in limited cases of posteriorly displaced physeal fractures without TOS [5,6]. This is because remodeling occurs along an intact periosteal sleeve as the physis ossifies. A similar mechanism allows non-operative management of childhood acromioclavicular joint fracture-dislocations at the lateral clavicle [21]. Conservative management avoids exposure to operative risk and the overtreatment of patients. It may be suitable for select patients with or without TOS depending on the degree of joint displacement, absence of radiographic compression on CT and a patient’s suitability for surgery.

## 4 Conclusion

Recognising posterior sternoclavicular joint dislocation is critical because it is associated with potentially life-threatening injuries. Our case demonstrates successful outcomes of conservatively managed posterior sternoclavicular joint dislocation with associated venous and neurological thoracic outlet syndrome after 3 years. While advocating for the early open reduction of these injuries in many cases, we highlight that certain circumstances may influence the decision to forgo surgical intervention. This information may benefit select patients who are unsuitable or hesitant about surgery.

## Declaration of Competing Interest

The authors have no conflicts of interest to declare.

## Sources of funding

This research did not receive any specific grant from funding agencies in the public, commercial, or not-for-profit sectors.

## Ethical approval

Ethical approval was not required and patient-identifying information was not presented in this report.

## Consent

Written informed consent was obtained from the patient for publication of this case report and accompanying images. A copy of the written consent is available for review by the Editor-in-Chief of this journal on request.

## Author contribution

**Timothy McAleese:** Conceptualisation, data curation, methodology, visualisation, project administration, software, writing – original draft

**Mark Curtin:** Conceptualisation and design, Project administration, writing – review and editing.

**Denis Collins:** Supervision, conceptualisation, validation, writing – review and editing.

All authors read and approved the final manuscript.

## Registration of research studies

This is not applicable due to the nature of this case report.

## Guarantor

Mr Denis Collins is a consultant orthopaedic surgeon and the senior author in this paper. As guarantor he is responsible for the work and/or the conduct of the study, had access to the data and controlled the decision to publish.

## Provenance and peer review

Not commissioned, externally peer-reviewed.
